# A population-based gene expression signature of molecular clock phase from a single epidermal sample

**DOI:** 10.1186/s13073-020-00768-9

**Published:** 2020-08-21

**Authors:** Gang Wu, Marc D. Ruben, Lauren J. Francey, David F. Smith, Joseph D. Sherrill, John E. Oblong, Kevin J. Mills, John B. Hogenesch

**Affiliations:** 1 Divisions of Human Genetics and Immunobiology, Center for Circadian Medicine, Department of Pediatrics, Cincinnati Children’s Hospital Medical Center, 240 Albert Sabin Way, Cincinnati, OH 45229 USA; 2 Divisions of Pediatric Otolaryngology, Pulmonary Medicine, and the Sleep Center, Cincinnati Children’s Hospital Medical Center, 3333 Burnet Ave, Cincinnati, OH 45229 USA; 3 Department of Otolaryngology-Head and Neck Surgery, University of Cincinnati College of Medicine, 231 Albert Sabin Way, Cincinnati, OH 45267 USA; 4 The Procter and Gamble Company, Mason Business Center, 8700 Mason Montgomery Road, Mason, OH 45040 USA

**Keywords:** Skin, Dermis, Epidermis, Circadian medicine, Population rhythm, Circadian biomarkers

## Abstract

**Background:**

For circadian medicine to influence health, such as when to take a drug or undergo a procedure, a biomarker of molecular clock phase is required––one that is easily measured and generalizable across a broad population. It is not clear that any circadian biomarker yet satisfies these criteria.

**Methods:**

We analyzed 24-h molecular rhythms in human dermis and epidermis at three distinct body sites, leveraging both longitudinal (*n* = 20) and population (*n* = 154) data. We applied cyclic ordering by periodic structure (CYCLOPS) to order the population samples where biopsy time was not recorded. With CYCLOPS-predicted phases, we used ZeitZeiger to discover potential biomarkers of clock phase.

**Results:**

Circadian clock function was strongest in the epidermis, regardless of body site. We identified a 12-gene expression signature that reported molecular clock phase to within 3 h (mean error = 2.5 h) from a single sample of epidermis––the skin’s most superficial layer. This set performed well across body sites, ages, sexes, and detection platforms.

**Conclusions:**

This research shows that the clock in epidermis is more robust than dermis regardless of body site. To encourage ongoing validation of this putative biomarker in diverse populations, diseases, and experimental designs, we developed SkinPhaser––a user-friendly app to test biomarker performance in datasets (https://github.com/gangwug/SkinPhaser).

## Background

In the last 50 years, dozens of clinical studies showed that dosing time-of-day can impact the efficacy and safety of many different types of medical treatments [1, 2]. We know from multi-tissue studies in mice [3] and humans [4] that thousands of rhythmically expressed genes encode known drug targets. Circadian medicine aims to incorporate this knowledge to improve treatment outcomes. This requires a reliable measure of a patient’s molecular clock phase as wall time does not necessarily equate to clock gene phase. Accumulating evidence shows interpersonal variation in the timing of physiology and behavior due to genetics, age, sex, and lifestyle [5–8]. Robust markers of the molecular clock phase are therefore in high demand.

Previous research focused on dim-light melatonin-onset (DLMO) as a marker of suprachiasmatic nucleus (SCN) phase [9, 10]. However, DLMO is inconvenient, costly, difficult to standardize, and thus rarely used clinically. With the development of high-throughput molecular detection platforms and computational techniques, recent efforts shifted to machine learning predictions based on the transcriptome or metabolome from one or two samples of whole blood [11–16], or a specific blood cell type (e.g., CD14^+^ monocytes) [17]. This approach can predict DLMO phase to within 3 h from a single blood sample [14, 17]. However, studies were small (≤ 74 participants) and limited to younger participants (18–41 years of age). Developing and validating blood-based biomarkers of DLMO phase in the broader population remain a challenge. Additionally, it is not clear what the melatonin phase tells us about clock gene phase in other tissues.

Previously, we showed human skin has a more robust circadian clock than blood, and developed a 29-gene expression signature from ~ 300 human forearm epidermis samples. From a single biopsy, this set estimated molecular clock phase in epidermis to within 3 h [18]. However, for clinical use, critical questions remain such as whether this expression signature performs across different skin locations, in different populations, and on different platforms.

Using an experimental design that captures the advantages of both longitudinal and population-based studies, we analyzed 24-h molecular rhythms in human dermis and epidermis at three distinct body sites. Participants in the longitudinal group (*n* = 20, aged 21–49 years) each donated one skin punch biopsy every 6 h across a day. Participants in the population group (*n* = 154, aged 20–74 years) each donated one punch biopsy from the forearm, buttock, and cheek without recording time. For all biopsies, the dermis was separated from the epidermis by laser capture microdissection (LCM) and then profiled on gene expression arrays. We applied cyclic ordering by periodic structure (CYCLOPS) [19] to order the population samples where biopsy time was not recorded.

We found that circadian clock function was strongest in the epidermis, regardless of body site. Based on this, we applied ZeitZeiger [20] and identified a potential biomarker set from a single epidermal sample that reported molecular clock phase in the skin to within 3 h. This set performed well across body sites, ages, sexes, and detection platforms and represents a forward path to clinical application of circadian medicine.

## Methods

### Collection of human longitudinal dermis samples

Twenty healthy Caucasian male participants from the USA were recruited for the longitudinal dermis sample collection. These were the same 20 participants in our previous collection of longitudinal epidermis samples [18]. All participants provided informed consent, and the associated protocol was approved by an Institutional Review Board (Aspire; http://aspire-irb.com/). Participants donated one skin punch biopsy at each of four time points (6 AM, 12 PM, 6 PM, and 12 AM) over a 24-h period. Biopsies were separated into epidermal and dermal layers by LCM. The mRNA extraction, target labeling, and hybridization to microarrays were described previously [21].

### MetaCycle analysis of longitudinal data

The RMA algorithm from the Affy R package [22] was used to extract the expression profile from the raw CEL files of 79 human longitudinal arm dermis samples (Additional file 1: Table S1). Expression profiles were analyzed with the meta3d function of MetaCycle (Additional file 1: Table S2) R package [23] using default settings, except “cycMethodOne” = “ARS,” “minper” = 24, and “maxper” = 24. ARSER [24] was used to analyze the time-series data individual by individual. Then, meta3d was used to integrate the analysis results from 19 participants (participant 115 had one missing time point and was excluded from the longitudinal analysis). Circadian genes were defined by *P* < 0.05 and relative amplitude (rAMP) > 0.1. We applied a less strict cutoff (*P* < 0.1 and rAMP > 0.1) for evaluating overlap between human epidermis and dermis, and phase set enrichment analysis (PSEA) [25]. Salivary melatonin and cortisol were also measured every 3 h over 24 h for 16 of 20 participants (participants 101, 102, 103, and 104 only have 3 or 4 measures) [18]. MetaCycle’s meta2d function predicted melatonin and cortisol phases (Additional file 2: Table S3) for these 16 participants using default settings, except “minper” = 24 and “maxper” = 24. We note that the relatively low sampling resolutions of skin biopsy and salivary collections reduce the accuracy of MetaCycle phase predictions.

### Comparing clock robustness between human epidermis and dermis

We used the microarray dataset from a previous population study of human epidermis and dermis (Additional file 1: Table S1) [21]. This study recruited 154 Caucasian females (aged 20–74 years) in the USA. Three punch biopsies were collected from each participant, with one biopsy per body site (arm, buttock, and cheek). Sample collection times were not recorded. Epidermal and dermal layers were separated by LCM. This yielded six groups of skin samples: arm epidermis, buttock epidermis, cheek epidermis, arm dermis, buttock dermis, and cheek dermis. We measured pairwise clock gene correlations [26] between epidermis and dermis at each body site. A reference correlation matrix of 17 mouse clock and clock-associated genes was computed from 12 mouse tissues [3]. The correlation matrix of 17 human homolog genes was computed for each skin group. To evaluate clock robustness in each skin group, we computed a Mantel test statistic between the reference correlation matrix and the correlation matrix from each skin group.

### The hybrid design and seed circadian gene lists

We used the hybrid design to order epidermis and dermis samples across body sites [18]. In detail, the RMA algorithm was applied to all 533 epidermis samples (79 longitudinal samples from the arm, and all population-based samples from the arm, buttock, and cheek except 8 missing samples). Data were ComBat [27] corrected for batch effects. Probe sets were annotated with gene symbols, and one representative probe set with the maximum median absolute deviation was selected for each gene. These same steps were performed for 531 dermis samples (79 longitudinal samples from the arm, and all population-based samples from the arm, buttock, and cheek except 10 missing samples).

### Ordering human epidermal and dermal samples using the revised CYCLOPS pipeline

The revised CYCLOPS pipeline (Additional file 1: Table S2) [18, 19] was used to order human epidermal and dermal samples. Prior to ordering, we added a down-sampling step [4]. The sample list with the maximum clock oscillation signal was kept for CYCLOPS ordering after serially sampling 5 million random selections. We included 97% (95%) of samples from all epidermis (dermis). Maximum clock oscillation signal was defined as the highest *Z*-statistic (Mantel test) compared to the benchmark correlation matrix (Additional file 3: Table S4) of 298 previously ordered human epidermis samples [18]. In sum, 519 (~ 97%) of epidermis samples and 506 (~ 95%) of dermis samples were selected for CYCLOPS ordering.

To further optimize CYCLOPS ordering quality, we tested three different seed lists as input to the algorithm: (1) 158 genes that are identified as circadian genes in at least two of four longitudinal skin datasets—human dermis from this study, human epidermis [18], and mouse time-series telogen and anagen [28]; (2) human homologs of genes cycling in at least 9 of 12 mouse tissues [3]; and (3) 17 clock and clock-associated genes (Additional file 4: Table S5). Assessment of ordering quality was based on two Fisher circular correlation values: (1) sampling time correlation value and (2) clock gene correlation value. For epidermis, best results were obtained from 519 samples ordered using seed set #3 (above). For dermis, best results were obtained from 506 samples ordered using seed set #2. Circadian genes were selected with FDR < 0.05, fitmean > 16, rAMP > 0.1, and goodness-of-fit (rsq) > 0.1 from the cosinor regression analysis results of matched epidermis and dermis samples with CYCLOPS-predicted phases. The phase was adjusted with *ARNTL* (*Arntl*) phase for phase comparison between human and mouse skin for those circadian genes identified in human skin with a cycling homolog gene in mouse telogen. The circadian genes identified in mouse telogen were the same as the previous analysis [18] of time-series mouse telogen data [28].

### ZeitZeiger to identify potential biomarkers of molecular clock phase

ZeitZeiger [20] was used to identify potential biomarkers of molecular clock phase from CYCLOPS-ordered human epidermis and dermis samples. Epidermis or dermis samples were divided into two groups: testing and training. The testing set in epidermis (and dermis) included 36 time-stamped samples from nine participants (each participant with four samples). The epidermis (and dermis) training set included all ordered samples from the population group and from the remaining 11 participants of the longitudinal group. In total, 483 epidermis samples and 472 dermis samples were used for training ZeitZeiger. Default ZeitZeiger settings were used for training samples, with two modifications: (1) normalizing the expression value of each gene and (2) selecting “dynamic genes” with large daily expression variation. There are three steps in expression normalization: (1) normalizing by three non-circadian genes in the skin (*GPKOW*, *BMS1*, and *ANKFY1*), (2) rounding the expression outliers, and (3) normalizing the expression value of each gene using its maximum expression value across all samples. “Dynamic genes” were defined by an *R*-squared value > 0.1 from cosinor regression analysis of the optimal CYCLOPS ordering of epidermis and dermis samples. We ran ZeitZeiger using sumabsv = 2 and sumabsv = 3 in epidermis and dermis, respectively. Those genes in the first two SPCs with an absolute coefficient value > 0.05 were selected as candidate biomarkers of molecular clock phase.

### Validation of skin phase prediction on different longitudinal datasets

The prediction accuracy of 12 epidermal or 21 dermal genes was further evaluated using the 36 time-stamped epidermis or dermis samples in the testing set. The phase difference between any two time-stamped samples for the same participant is known and is not influenced by the individual chronotype. The prediction accuracy is thus evaluated by comparing the difference between predicted and known sample phases.

The potential biomarker set was then tested on an independent longitudinal dataset [29] of 20 Caucasian male and female participants in Germany. In this study, epidermis samples were harvested at 9:30 AM, 2:30 PM, and 7:30 PM using suction-blister method, with transcriptome detection on an Agilent platform. Eighteen participants each with 3 epidermis samples were used for testing the prediction accuracy of our potential biomarker.

### Statistical analysis

All statistical analyses were performed in R. Relevant packages are listed in Additional file 1: Table S2. The two-tailed Mantel test was used to test the similarity between correlation matrices. The two-tailed Wilcoxon test was used to test for amplitude differences in rhythmic gene expression. Statistical cutoffs of *P* < 0.05 were used to define “circadian genes,” unless otherwise noted. Fisher’s circular correlation value was used to compare (1) CYCLOPS-predicted versus known sample phases, and (2) clock gene phases in human versus mouse. The Benjamini-Hochberg correction (FDR < 0.05) was used for selecting circadian genes from population-level analysis of human epidermis and dermis across body sites.

## Results

### A functional clock in the human dermis

To explore molecular rhythms in human dermis, forearm skin samples were collected from the longitudinal group every 6 h from 20 individuals (participant 115 had one missing time point and was excluded; Additional file 1: Table S1). The dermal layer was separated from the epidermal layer using LCM. Using MetaCycle [23], we identified 182 circadian genes (*P* < 0.05; Additional file 1: Fig. S1A) in the dermis. The distribution was bimodal (Additional file 1: Fig. S1B) with peak phases clustered at 8–9 AM and 8–9 PM, as previously seen in epidermis [18] and other human tissues [30–32]. Morning-time genes were enriched for immune-related and G protein-coupled receptor (GPCR) signaling, whereas the evening was marked by genes involved in transcription, lipid and lipoprotein metabolism, and development (Additional file 1: Fig. S1C-D) (phase set enrichment analysis (PSEA) [25]).

### Clock strength varies between skin layers

**Fig. 1:**
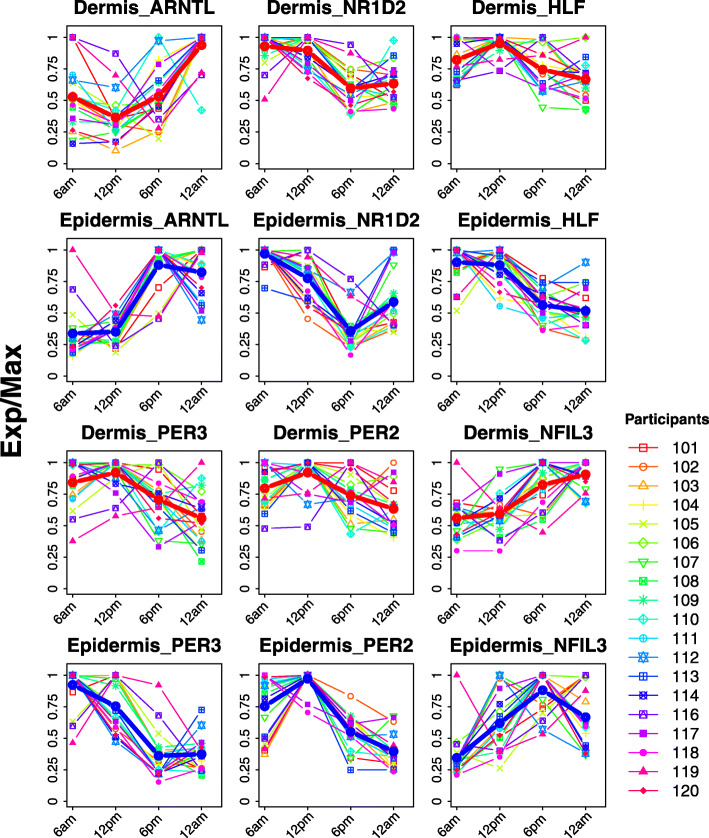
Expression profiles of overlapped clock and clock-associated genes in human dermis and epidermis. Forearm punch biopsies were collected every 6 h for 1 day starting at 6 AM. Expression profiles from 19 participants over time are shown for dermis (rows 1 and 3) and epidermis (rows 2 and 4). Red and blue lines represent the average expression profile among all participants for dermis and epidermis, respectively. Exp/Max indicates the expression value at each time point normalized to the maximum expression across four time points

We next compared clock gene oscillations between forearm dermis and epidermis. The peak phases of expression for six clock genes (*ARNTL*, *NR1D2*, *HLF*, *PER3*, *PER2*, and *NFIL3*; *P* < 0.1) (Fig. 1) between skin layers were consistent in participants. For example, the median phase difference between epidermis and dermis samples is 0.4 h for *ARNTL* and 0.6 h for *NR1D2* (Additional file 1: Fig. S1E). In addition, *ARNTL* peaked ~ 7 h before melatonin in both the epidermis and dermis layers (Additional file 1: Fig. S2).

Although clock gene phases were similar between skin layers, the strength of their oscillations was not. For each of the six clock genes that were rhythmic in both layers, amplitudes were higher in the epidermis. For example, the median rAMP of *PER3* was twofold greater in the epidermis compared to dermis (0.59 vs. 0.29, *P* = 1.9e−6) (Additional file 1: Fig. S1F).

**Fig. 2:**
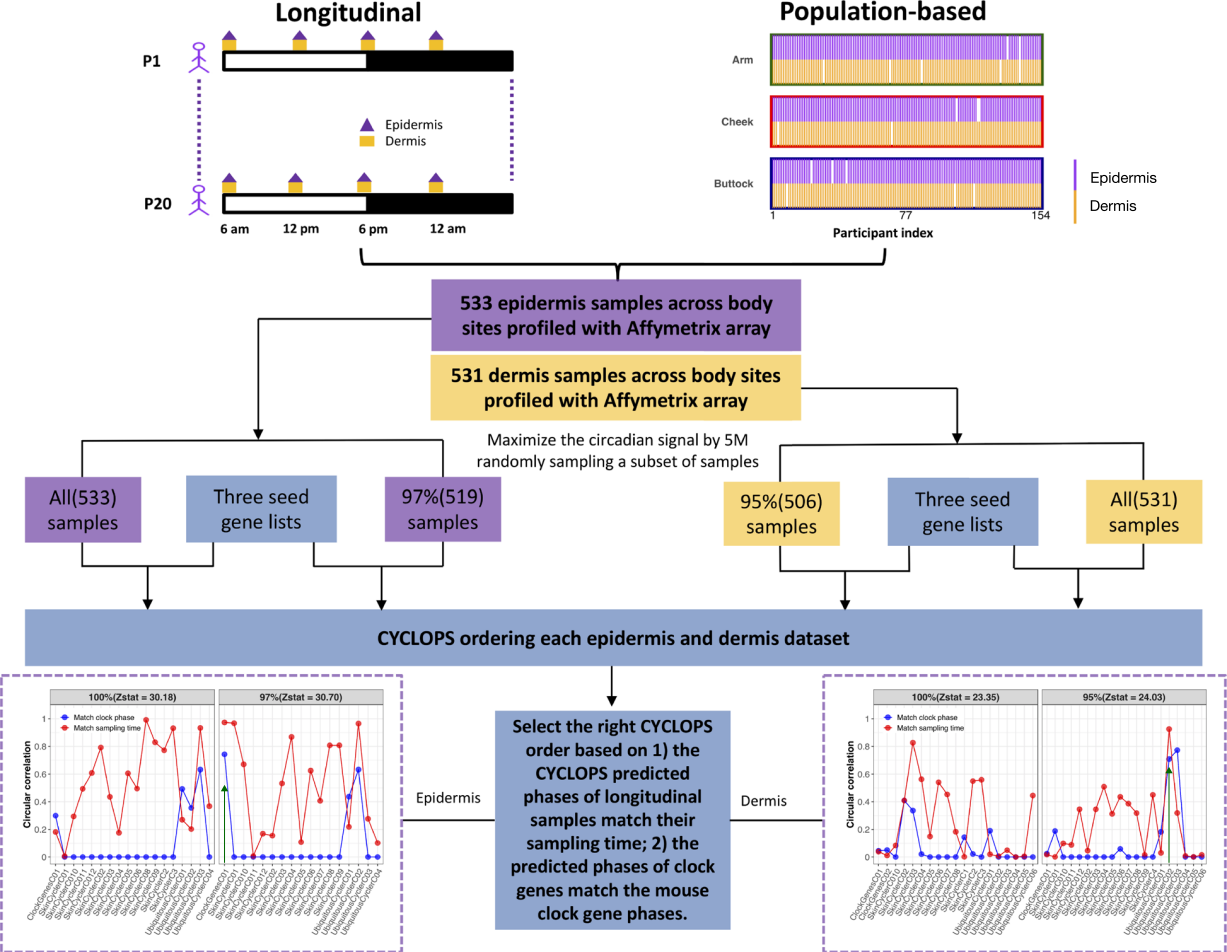
The pipeline for CYCLOPS ordering in epidermis and dermis samples. Each skin layer has two datasets. One dataset includes all epidermis (dermis) samples by combining longitudinal samples collected from 20 participants and population-based samples collected from 154 participants at three body sites (arm, cheek, and buttock). Another dataset has the maximum circadian signal in 5 million random subsetting 97% (95%) samples from all epidermis (dermis) samples. For CYCLOPS ordering these four datasets, we used three seed circadian gene lists, including (1) skin circadian genes, (2) human homologs of mouse ubiquitous circadian genes, and (3) clock and clock-associated genes (Additional file 4: Table S5). The high-quality CYCLOPS order should match both sampling time and clock phase. The matchability of sampling time and clock phase was measured by the corresponding circular correlation value. If fewer than 8 clock genes satisfied the cutoff (*P* < 0.01, fitmean > 16, rAMP > 0.1, and rsq > 0.1), the circular correlation value for “match clock phase” was set as 0. The optimal CYCLOPS order was indicated with a green arrow, with both high circular correlation values of sampling time and clock phase

**Fig. 3:**
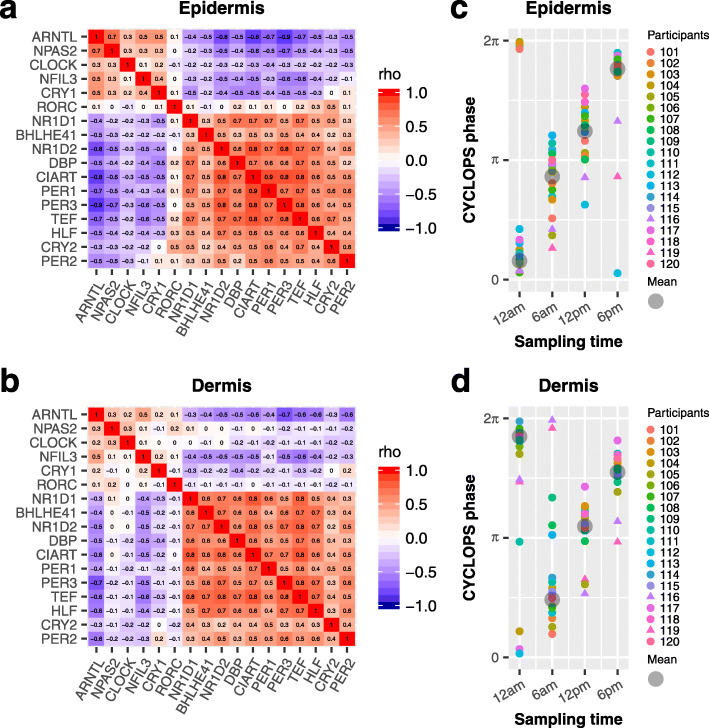
Evaluation of clock function and accuracy of CYCLOPS ordering in epidermis and dermis. A heatmap of Spearman’s rho for clock and clock-associated genes from longitudinal (*n* = 20, Additional file 1: Table S1) and population-based data (*n* = 154, Additional file 1: Table S1) is shown for epidermis (**a**) and dermis (**b**). The population-based epidermis or dermis samples are from the forearm, cheek, and buttock of the same 154 participants. The longitudinal order of samples collected from 20 participants was accurately recalled in epidermis (**c**) and dermis (**d**). Different colors indicate different participants, and the circular average phases for all samples are shown in gray at each sampling time. Samples from participants 116 and 119 are indicated by triangles

To evaluate clock function in the broader population, we next analyzed clock gene expression from 154 participants, aged 20–74 years. Each participant contributed three punch biopsies from the cheek, forearm, and buttock [21]; sampling time was not recorded (Fig. 2). To compare robustness of clock oscillations between different skin layers and body sites, we computed correlation matrices of clock and clock-associated genes [26] for each of the six conditions (e.g., epidermis-cheek, dermis-cheek). Each of these matrices was compared to a reference matrix constructed from 12 mouse tissues (Additional file 5: Table S6). To quantify this, a Mantel test *Z*-statistic was computed, which provided a measure of similarity between the mouse reference and each of the six conditions. Larger values indicate stronger clock oscillation. As expected, forearm epidermis (*Z*-statistic = 33.3; Additional file 1: Fig. S3A) was stronger than dermis (*Z*-statistic = 29.6; Additional file 1: Fig. S3B). This was also true in the non-sun-exposed buttock, with a larger *Z*-statistic value in epidermis (*Z*-statistic = 34.7; Additional file 1: Fig. S3C) than dermis (*Z*-statistic = 32.1; Additional file 1: Fig. S3D). There was no obvious difference in clock robustness between cheek epidermis (*Z*-statistic = 35.3; Additional file 1: Fig. S3E) and dermis (*Z*-statistic = 35.6; Additional file 1: Fig. S3F). When combining all longitudinal and population study samples from three body sites, the epidermis (*Z*-statistic = 37.6; Fig. 3a) was stronger than dermis (*Z*-statistic = 32.5; Fig. 3b). Of note, although the dermal clock is weaker, it is still functional (Mantel test *P* = 5.0e−06).

### Genome-wide rhythms in epidermis and dermis across body sites

Based on evidence of functional clocks in epidermis and dermis, we explored their rhythmic transcriptomes. Because biopsy time was not recorded for any of the population samples, we used CYCLOPS to reconstruct their temporal order, as reported previously [18]. Samples from epidermis and dermis were ordered separately. To maximize the oscillation signal, we added a random sampling step (Fig. 2, detailed in the “Methods” section). Using this strategy, we achieved high-quality ordering with 97% (epidermis) and 95% (dermis) samples. Assessment of ordering quality was based on two primary criteria: (1) CYCLOPS-predicted sampling times for the 20 participants with longitudinal data matched *known* sampling times (Fisher’s circular correlation of 0.973 in epidermis and 0.925 in dermis for averaged CYCLOPS phase at each of four time points) (Fig. 3c, d), and (2) the predicted phases of clock genes matched the mouse reference (Additional file 1: Fig. S4A-B, outer vs. inner circle; Fisher’s circular correlation, 0.74 in epidermis and 0.71 in dermis). In addition, predicted *ARNTL* phases correlated with known melatonin phases (Fisher’s circular correlation, 0.63 in epidermis and 0.54 in dermis) and cortisol phases (Fisher’s circular correlation, 0.62 in epidermis and 0.63 in dermis) of longitudinal samples (Additional file 1: Fig. S5).

**Fig. 4:**
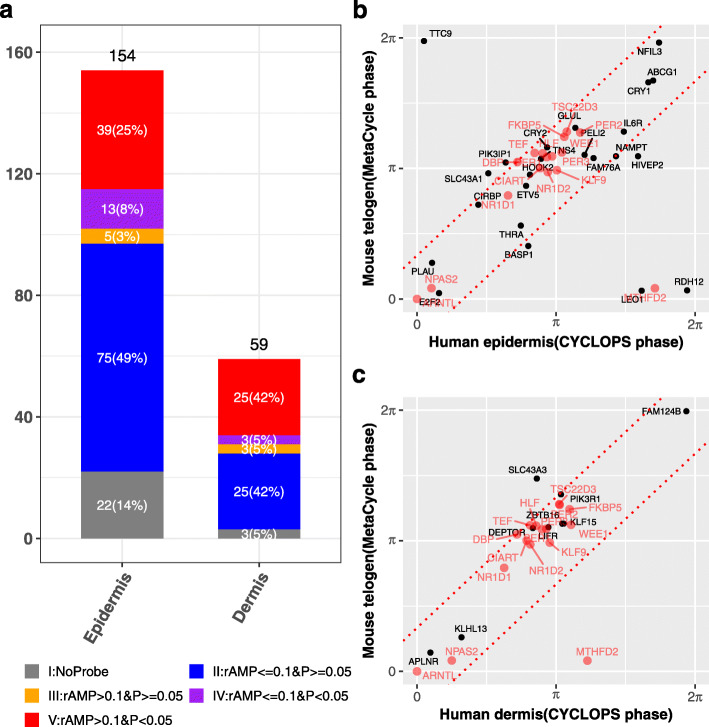
Conserved circadian transcriptional output between human and mouse skin. **a** Circadian genes in human epidermis and dermis were grouped into 5 categories: (I) no mouse homolog (gray), (II) low amplitude (rAMP ≤ 0.1) and not cycling (*P* > 0.05) (blue), (III) high amplitude (rAMP > 0.1) and not cycling (orange), (IV) low amplitude and cycling (*P* < 0.05) (purple), and (V) high amplitude and cycling (red). **b**, **c** Phase comparison of high amplitude cycling genes (group V, *n* = 39 for epidermis and *n* = 25 for dermis) reveals strong phase conservation between mice and humans. Overlapped circadian genes in epidermis and dermis are shown in red. The gene phases were adjusted to *ARNTL/Arntl* phase (set to 0). The red dotted line indicates the phase difference with π/3. Mouse data are from Geyfman et al. [28] (Additional file 1: Table S1)

We then analyzed 24-h patterns of expression for all transcripts genome-wide as a function of predicted sample phase. One hundred fifty-four and 59 genes met our criteria for rhythmicity in epidermis and dermis, respectively (modified cosinor regression, FDR < 0.05, rAMP > 0.1, rsq > 0.1, and fitmean > 16) (Fig. 4a, Additional file 6: Table S7). Thirty-nine (25%) of these genes from epidermis and 25 (42%) genes from dermis had robustly cycling homologs in mouse telogen, suggesting evolutionary conservation. Mouse telogen refers to the non-proliferative stage in the skin, which has stronger circadian output than the hair follicle growth stage [28]. Most of these conserved genes (Fig. 4b, c) oscillated with similar phases (relative to *ARNTL*/*Arntl* phase) between species (within 4 h difference). Sixteen of them were rhythmically expressed in *both* epidermis and dermis, 11 of which were clock and clock-associated genes. Interestingly, 4 of the 5 remaining genes (*WEE1*, *TSC22D3*, *FKBP5*, and *KLF9*), although not currently considered clock-associated, were circadian-expressed in many mouse tissues (JTK *Q* value < 0.05 in 7–13 mouse tissues from CircaDB) [33].

### The epidermal layer provides the better marker of skin phase

**Fig. 5:**
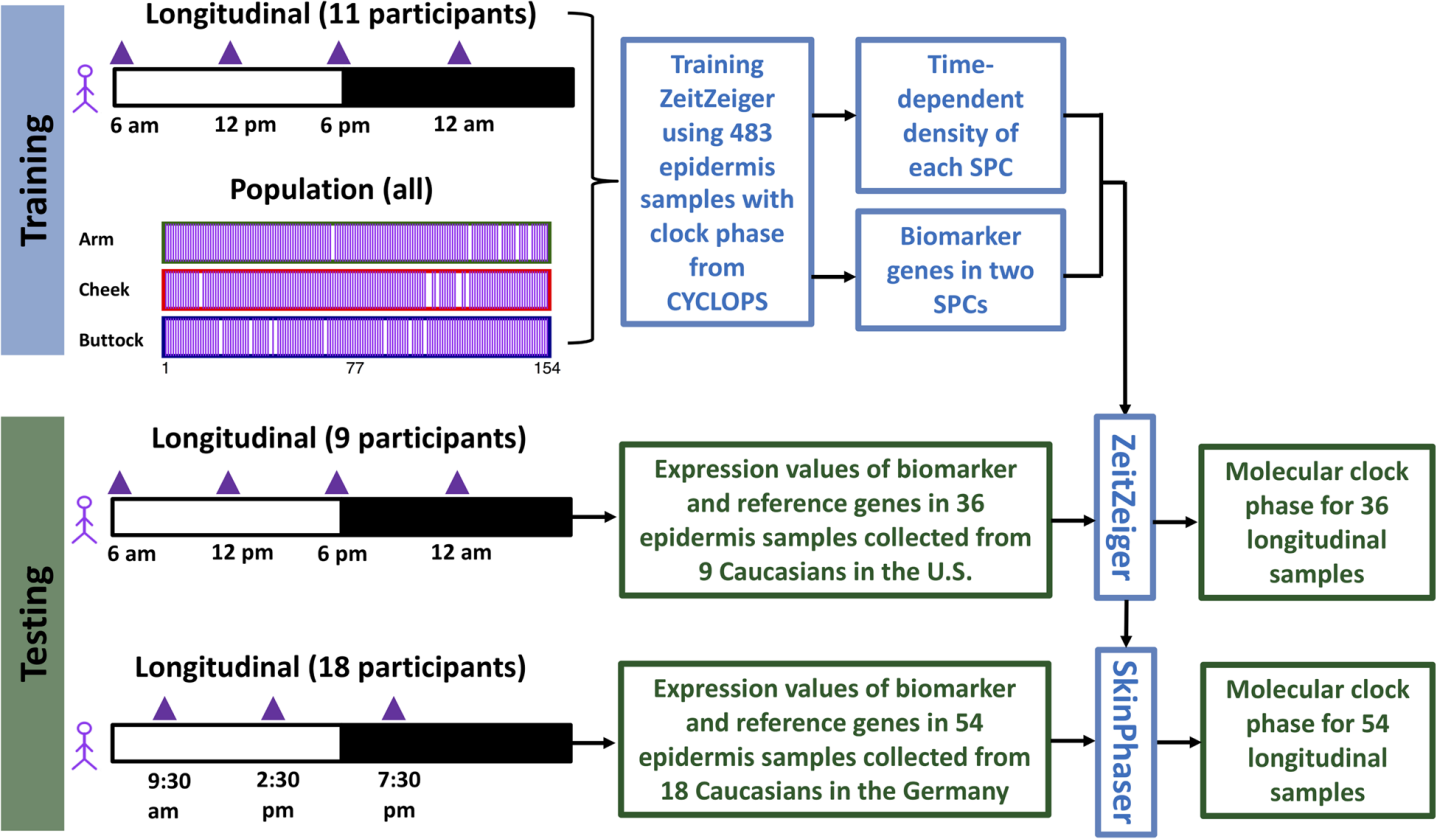
The pipeline for identifying and testing epidermal candidate biomarkers. The training dataset includes 483 longitudinal and population-based epidermis samples. Using this training dataset, a set of 12 candidate biomarker genes were selected by ZeitZeiger. The user-friendly SkinPhaser app was developed for predicting molecular clock phase using this set of 12 candidate biomarker genes. The prediction accuracy of candidate biomarkers was further tested using two testing datasets. One dataset includes 36 epidermis samples collected from 9 male participants in the USA, and each participant donated one sample at each of four time points (6 AM, 12 PM, 6 PM, and 12 AM). The other one includes 54 epidermis samples collected from 18 male and female participants in Germany, and each participant donated one epidermis sample at each of three time points (9:30 AM, 2:30 PM, and 7:30 PM)

**Fig. 6:**
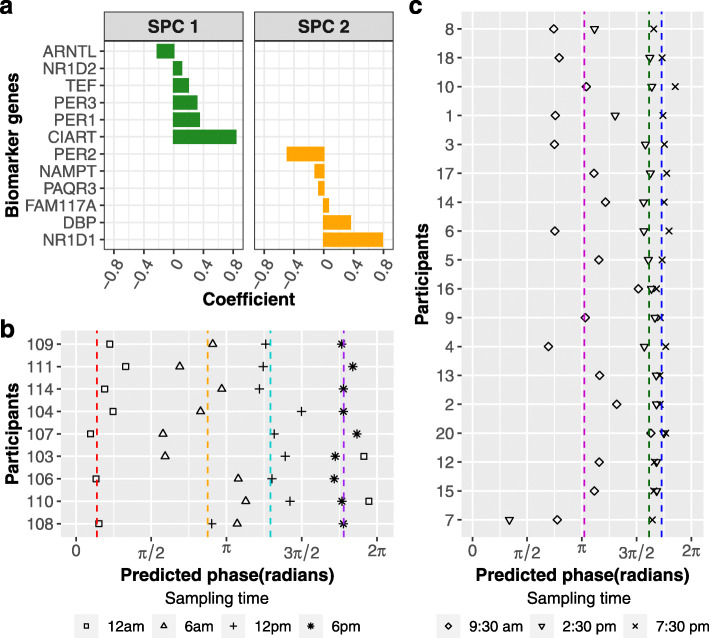
Population-level skin candidate biomarkers work for a single human epidermis sample. **a** Twelve marker genes in the first two sparse principal components (SPCs) were selected by ZeitZeiger. **b** Validation of skin candidate biomarkers using epidermis samples from 9 participants that were excluded from the training set. The epidermis samples were collected every 6 h over a circadian day. Average predicted phases of samples collected at 12 AM, 6 AM, 12 PM, and 6 PM are indicated with red, orange, cyan, and purple dashed lines, respectively. **c** Validation of skin candidate biomarkers using longitudinal epidermis samples from 18 participants in Spörl et al. [29] (Additional file 1: Table S1). The epidermis samples were collected every 5 h from 9:30 AM to 7:30 PM. Average predicted phases of samples collected at 9:30 AM, 2:30 PM, and 7:30 PM are indicated with magenta, green, and blue dashed lines, respectively. The predicted molecular clock phase is between 0 and 2π, with 0 indicating the peak phase of *ARNTL*

To evaluate which skin layer is the more reliable predictor of phase, we used ZeitZeiger [20] to identify candidate biomarker sets. ZeitZeiger was trained by a combination of population and longitudinal samples. There were two separate training datasets for CYCLOPS-ordered epidermis (*n* = 483) and dermis (*n* = 472) samples from the forearm, buttock, and cheek (Fig. 5, Additional file 1: Fig. S6). Training produced candidate biomarker sets of 12 and 21 genes for epidermis (Fig. 6a) and dermis (Additional file 1: Fig. S7A), respectively.

We tested the candidate biomarker sets in 36 epidermis and dermis samples from the same 9 participants. Each of these participants contributed 4 time-stamped samples that were not part of the training set. Whereas the epidermis set reproduced known sampling order in 8/9 participants, the dermis set only performed in 5/9 (Fig. 6b, Additional file 1: Fig. S7B). The predicted phases for epidermis and dermis samples collected in the early morning (6 AM) showed larger variation than other time points (Fig. 6b, Additional file 1: Fig. S7B). Overall, the average absolute error across different times of day for the 9 participants was 2.5 h (SD = 1.86 h) for epidermis compared to 3.8 h (SD = 3.92 h) for dermis (Additional file 1: Fig. S8A-B). In summary, the epidermis was the better source of biomarkers of skin phase than dermis.

### A skin clock candidate biomarker that is robust to a different sample collection method

We evaluated prediction accuracy in another dataset obtained from suction-blister epidermal samples collected over 24 h from 18 Caucasian participants in Germany [29] to test whether this candidate biomarker performs well with different sample collection methods. The candidate biomarker reproduced the time-stamped sample order in 14 of 18 participants (Fig. 6c). One sample phase in each of the four remaining individuals (participants 20, 12, 15, and 7) was poorly predicted. The overall average absolute error was 3.1 h (SD = 2.12 h, Additional file 1: Fig. S8C).

For future validation of this potential biomarker in diverse populations, diseases, and experimental designs, we developed SkinPhaser (https://github.com/gangwug/SkinPhaser) to enable the biomarker test (Additional file 1: Fig. S9) in public datasets.

## Discussion

We previously reported 29 genes from epidermal forearm skin whose expression values could determine skin phase to within 3 h [18]. The work here improves upon this and brings us closer to a clinically viable biomarker of molecular clock phase in human tissues.

First, a biomarker should be stable. We demonstrate this across multiple different body sites, sun- and non-sun-exposed. Second, a biomarker should be practical; its value must exceed its cost. We demonstrate a smaller set (12 genes) with improved accuracy from a single biopsy of the skin’s most superficial layer. Third, an ideal biomarker performs regardless of detection platform. We demonstrate this for two different array platforms (Agilent vs. Affymetrix) and collection strategies (suction blister vs. punch biopsy). Finally, a molecular clock biomarker should generalize to a diverse population; circadian medicine may apply to everyone. We demonstrate this for two different geographic groups (Germany and the USA). Nevertheless, testing of molecular clock biomarkers on a much broader range of datasets is critical. To help drive this forward, we developed SkinPhaser—a user-friendly app to benchmark this potential biomarker set across participant demographics, disease states, and experimental designs.

In both longitudinal and population datasets, molecular oscillations are more robust in epidermis than dermis. There are several possibilities associated with a stronger clock in epidermis than dermis. The dermal layer is much larger and more heterogeneous. Some cell types in the dermis may have weak intrinsic oscillators. Another possibility is that epidermal clocks may entrain more efficiently by virtue of their direct contact with environmental cues. Finally, systemic cues in the circulation may dampen dermal oscillations as the dermis is perfused, whereas the epidermis is not [34]. Nevertheless, despite differences in clock strength between epidermis and dermis, their phases of oscillation are similar and correlate with melatonin and cortisol phases. We conclude that epidermis is a better source of biomarkers of skin phase than dermis. We did not evaluate the whole skin, but speculate that its quality as an overall biomarker may be compromised by less robust layers, e.g., dermis. Similarly, CD14^+^ blood monocytes provide better prediction accuracy of DLMO phase than whole blood [17].

One important question is whether skin clock phase is a good predictor of clock phase in other tissues. In mice, the epidermal clock is under the control of the SCN [35], and levels of *Per2* in the skin were phase-aligned with levels in the liver in response to changes in food timing [36]. This suggests that skin phase *may* indicate phases of other tissues, including the liver––a key site for drug absorption, distribution, metabolism, and excretion. Time-of-day dosing of medications is one principal motivation for studying molecular clock biomarkers. Another important question is whether prediction “within 3 h” is sufficient for clinical application. This is likely to depend in part on drug kinetics. For drugs with a half-life longer than 6 h, “within 3 h” may be good enough. For short acting drugs or other applications (e.g., sleep and circadian disorders), better accuracy is needed. Future studies will need to link biomarker-predicted skin phases to physiology, and validate biomarker consistency across different experimental designs and disease states. Ultimately, clinical use of molecular clock biomarkers will require fast, cost-effective, non-invasive sampling, and an optimal detection platform. Finally, head-to-head comparisons are necessary to link the molecular clock phase predicted from skin biomarkers with the melatonin phase predicted from blood [14, 17] and other physiological phases (e.g., human rest/activity) [37].

With continuing efforts from multiple research groups in the last 30 years, the field is advancing towards robust and practical circadian biomarkers. Our particular goal is not to replace DLMO, but rather to develop a point of care test to optimize drug timing based on 24-h dynamics in drug absorption, metabolism, transport, excretion, and action.

## Conclusions

A functional clock running in human epidermis (12 candidate biomarker genes) is superior to dermis (21 candidate biomarker genes) to report molecular clock phase in the skin. Using one epidermis sample, this potential biomarker set performed well across body sites, ages, sexes, and detection platforms. We developed SkinPhaser—a user-friendly app to test these candidate biomarkers on community datasets.

## Supplementary information

**Additional file 1: Figure S1.** Comparison of circadian genes identified from longitudinal dermis and epidermis samples. **Figure S2.** MetaCycle predicted phases of *ARNTL*, melatonin and cortisol in epidermis and dermis for 20 participants. **Figure S3.** Evaluation of circadian clock robustness in epidermis and dermis across different body sites. **Figure S4.** Phase order of identified clock and clock-associated genes from epidermis and dermis. **Figure S5.** The relative phase to *ARNTL* matches relative phase to melatonin or cortisol for time-stamped samples in epidermis and dermis. **Figure S6.** The pipeline of identifying and testing dermal candidate biomarkers using ZeitZeiger. **Figure S7.** Candidate biomarkers for predicting molecular clock phase of a single dermal sample**. Figure S8.** Prediction accuracy of candidate biomarkers from epidermis and dermis. **Figure S9.** Three steps of running SkinPhaser. **Table S1.** The list of skin datasets used in this study. **Table S2.** The list of software packages used in this study.

**Additional file 2: Table S3.** Melatonin and cortisol phases predicted by MetaCycle.

**Additional file 3: Table S4.** The benchmark correlation matrix of 298 previously ordered human epidermis samples.

**Additional file 4: Table S5.** Seed gene lists used for CYCLOPS ordering.

**Additional file 5: Table S6.** The pairwise correlation matrix of 17 mouse clock and clock-associated genes.

**Additional file 6: Table S7.** Circadian genes identified from human epidermis and dermis.

## Data Availability

The gene expression data of longitudinal human dermis generated in this study are available at the Gene Expression Omnibus (GEO), GSE139300 [38]. The gene expression data of longitudinal epidermis were from GEO, GSE112660 [39] and GSE35635 [40]. The time-series expression data of mouse telogen were from GEO, GSE38622 [41]. The gene expression data of population epidermis and dermis were from GEO, GSE139305 [42]. The source code of SkinPhaser is available through Github: https://github.com/gangwug/SkinPhaser [43]. The software package versions used and their available links were listed in Additional file 1: Table S2.

## Abbreviations

CYCLOPS: Cyclic ordering by periodic structure
DLMO: Dim-light melatonin-onset
GPCR: G protein-coupled receptor
LCM: Laser capture microdissection
PSEA: Phase set enrichment analysis
