# Supplementary material for: A population-based gene expression signature of molecular clock phase from a single epidermal sample
Source: Genome Med. 2020 Aug 21;12:73. doi: 10.1186/s13073-020-00768-9 (PMC7441562; doi:10.1186/s13073-020-00768-9)
Supplement: Supplementary file 1 — Additional file 1: Figure S1. Comparison of circadian genes identified from longitudinal dermis and epidermis samples. Figure S2. MetaCycle predicted phases of ARNTL, melatonin and cortisol in epidermis and dermis for 20 participants. Figure S3. Evaluation of circadian clock robustness in epidermis and dermis across different body sites. Figure S4. Phase order of identified clock and clock-associated genes from epidermis and dermis. Figure S5. The relative phase to ARNTL matches relative phase to melatonin or cortisol for time-stamped samples in epidermis and dermis. Figure S6. The pipeline of identifying and testing dermal candidate biomarkers using ZeitZeiger. Figure S7. Candidate biomarkers for predicting molecular clock phase of a single dermal sample. Figure S8. Prediction accuracy of candidate biomarkers from epidermis and dermis. Figure S9. Three steps of running SkinPhaser. Table S1. The list of skin datasets used in this study. Table S2. The list of software packages used in this study. [file 13073_2020_768_MOESM1_ESM.docx]

**Additional file 1**

Fig. S1. Comparison of circadian genes identified from longitudinal dermis and epidermis samples.

Fig. S2. MetaCycle predicted phases of *ARNTL*, melatonin and cortisol in epidermis and dermis for 20 participants.

Fig. S3. Evaluation of circadian clock robustness in epidermis and dermis across different body sites.

Fig. S4. Phase order of identified clock and clock-associated genes from epidermis and dermis.

Fig. S5. The relative phase to *ARNTL* matches relative phase to melatonin or cortisol for time-stamped samples in epidermis and dermis.

Fig. S6. The pipeline of identifying and testing dermal candidate biomarkers using ZeitZeiger.

Fig. S7. Candidate biomarkers for predicting molecular clock phase of a single dermal sample.

Fig. S8. Prediction accuracy of candidate biomarkers from epidermis and dermis.

Fig. S9. Three steps of running SkinPhaser.

Table S1. The list of skin datasets used in this study.

Table S2. The list of software packages used in this study.


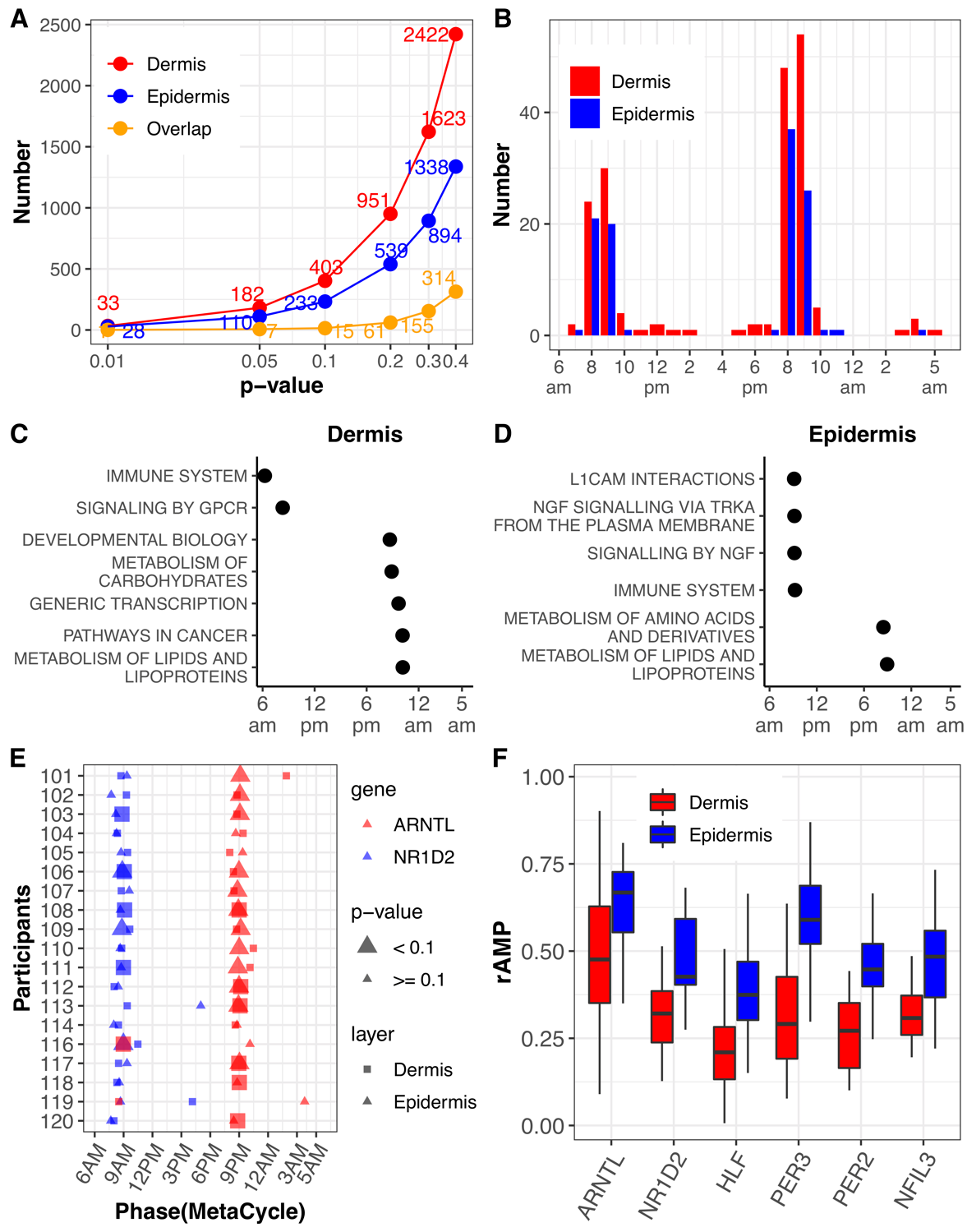


Fig. S1. Comparison of circadian genes identified from longitudinal dermis and epidermis samples. (A) Number of circadian genes at a series of p*-*value cut-offs were shown in dermis and epidermis. (B) Phase distribution of circadian genes (*P* < 0.05) identified in epidermis and dermis. (C and D) Significant enriched time-dependent pathways of circadian genes identified in dermis and epidermis. A less significant cut-off (*P* < 0.1) was used to select circadian genes for performing PSEA analysis (q < 0.05). (E) MetaCycle predicted phases of *ARNTL* and *NR1D2* in epidermis and dermis in 19 participants (excluding participant 115). (F) The individual rAMP of six clock genes were shown for dermis and epidermis. The boxes indicate data between the 25th and 75th percentiles with central horizontal lines representing the median values, respectively. The whiskers of boxes show the 5th and 95th percentiles.


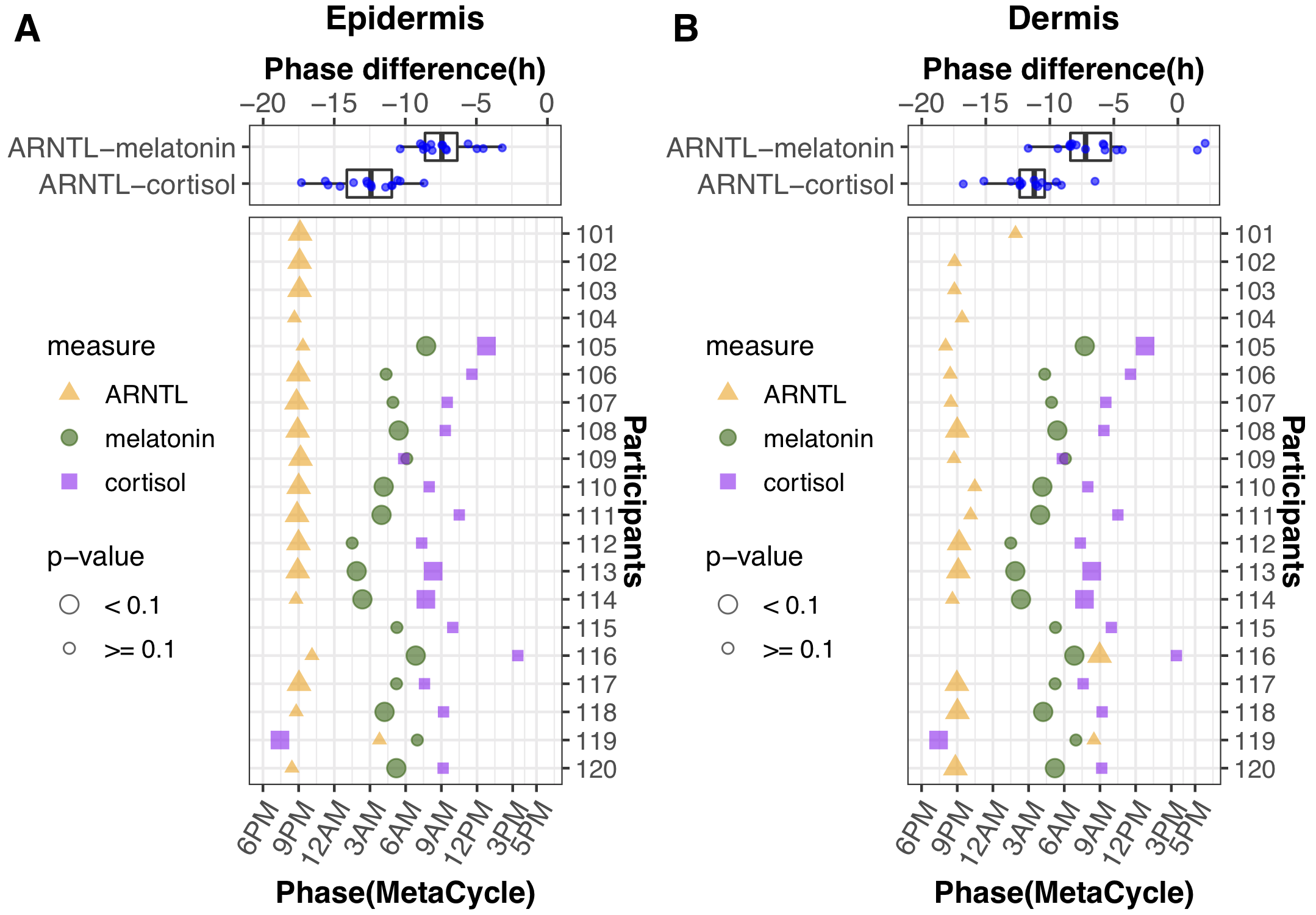


Fig. S2. MetaCycle predicted phases of *ARNTL*, melatonin and cortisol in epidermis and dermis for 20 participants. There are not enough data points for predicting melatonin and cortisol phase for participants 101, 102, 103 and 104, and for predicting *ARNTL* phase for participant 115. Total 15 participants have predicted *ARNTL*, melatonin and cortisol phases. The box-plots indicate the phase difference between *ARNTL* and melatonin/cortisol for these 15 participants in epidermis and dermis.


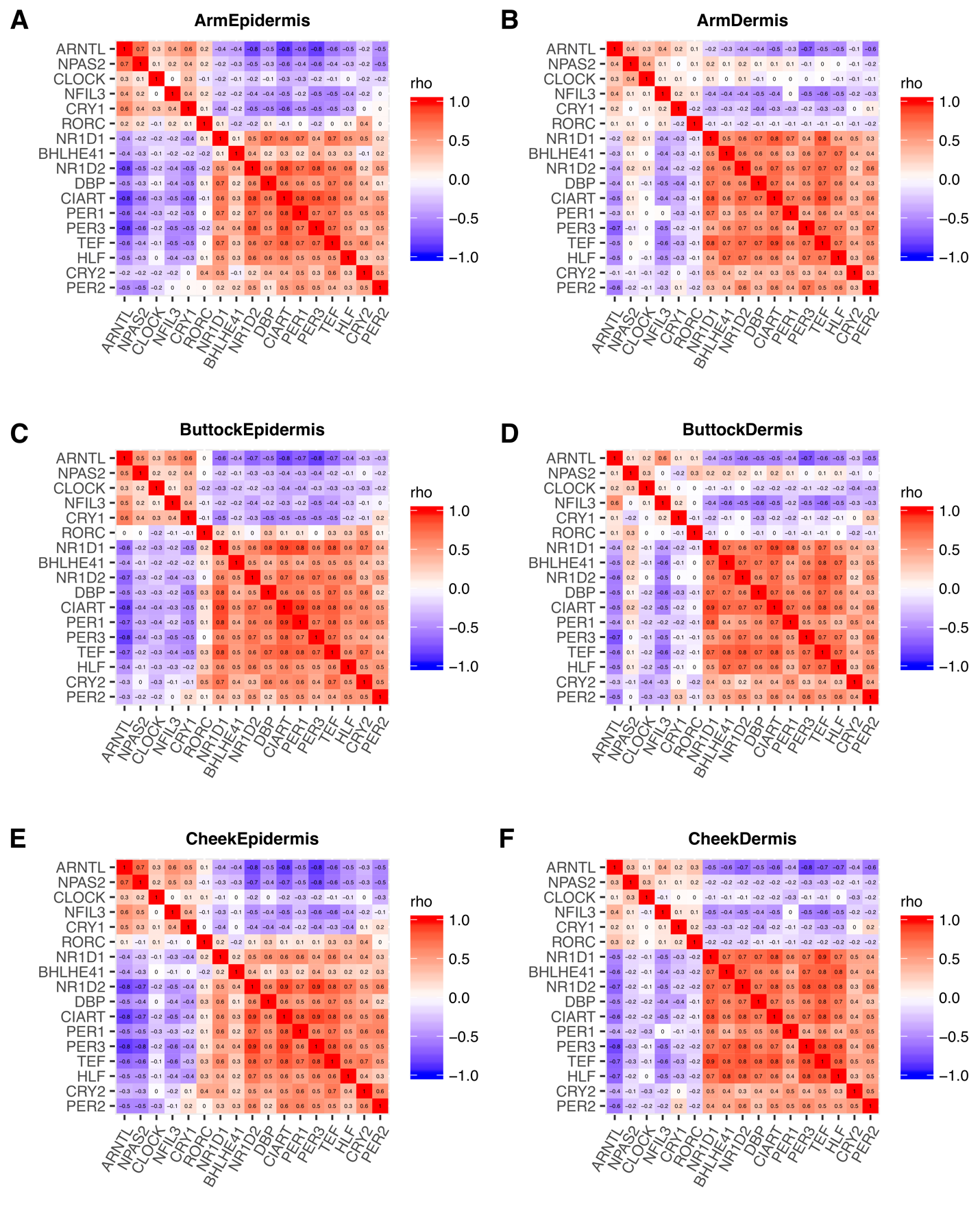


Fig. S3. Evaluation of circadian clock robustness in epidermis and dermis across different body sites. The heatmaps of Spearman’s rho correlation values for clock and clock-associated genes were drawn for epidermis (A, C, E) and dermis (B, D, F) in forearm, buttock and cheek for 154 participants. Red and blue indicate positive and negative Spearman’s rho values, respectively.


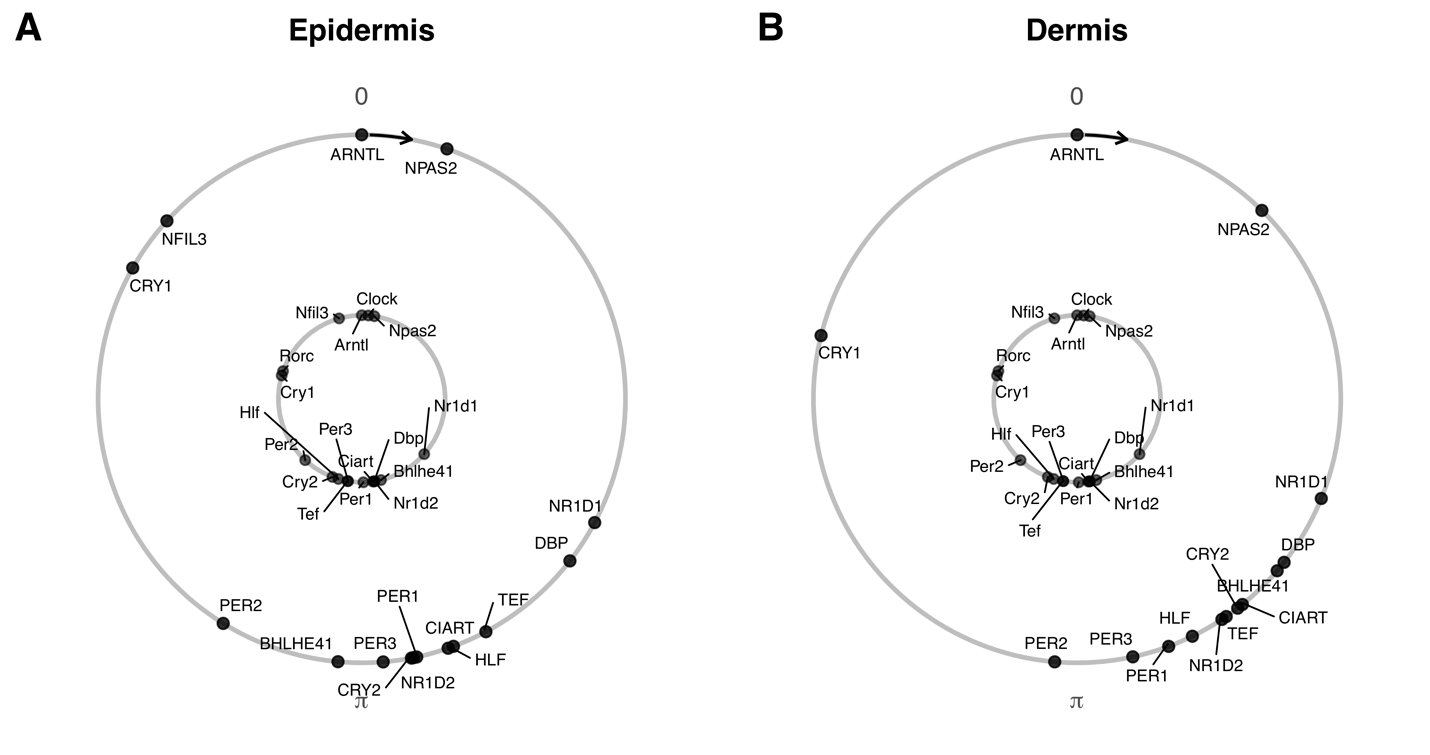


Fig. S4. Phase order of identified clock and clock-associated genes from epidermis and dermis. Conserved phase relationships are shown for clock and clock-associated genes (internal circle, mouse; external circle, human) in human epidermis (A) and dermis (B). The phase of *ARNTL/Arntl* is set as 0.


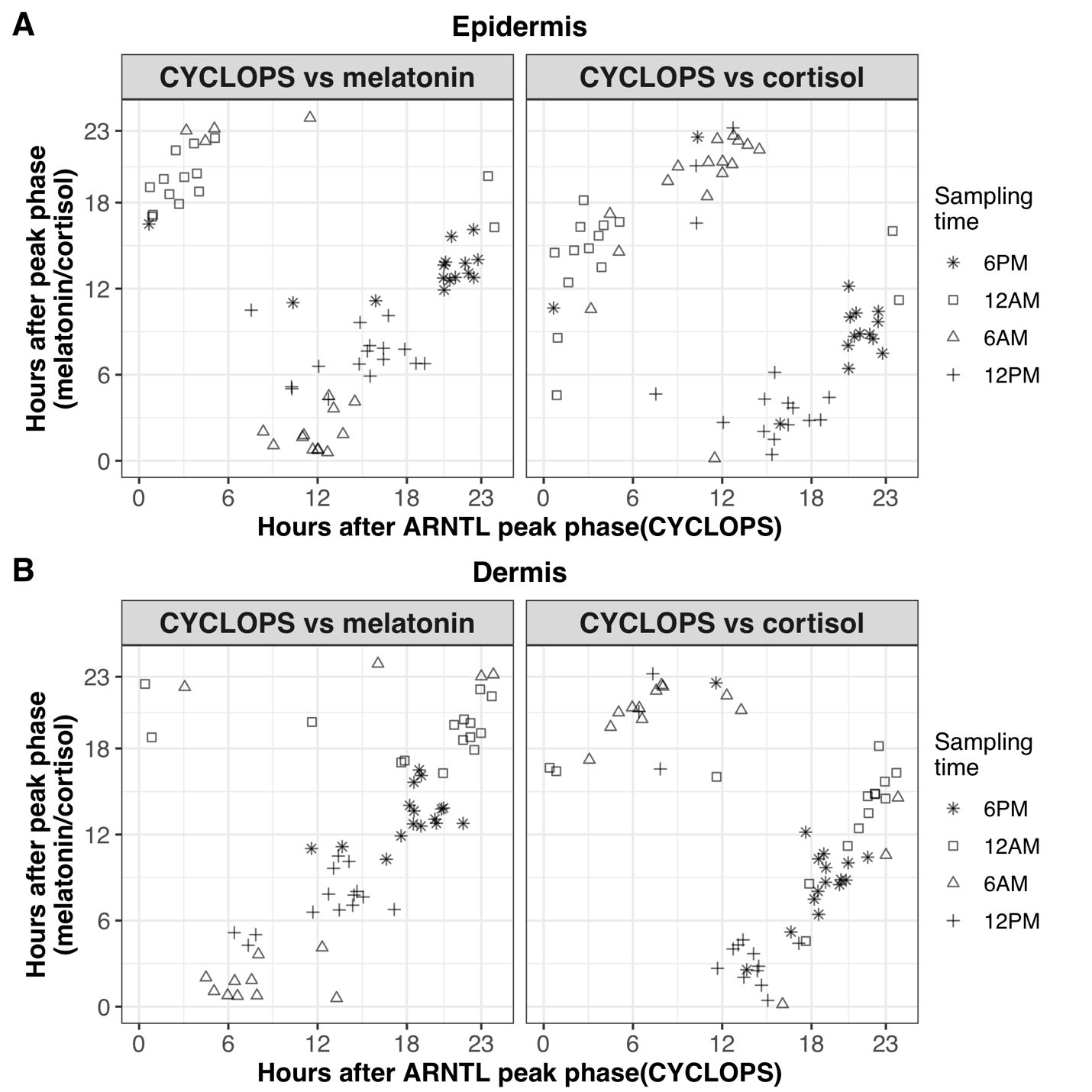


Fig. S5. The relative phase to *ARNTL* matches relative phase to melatonin or cortisol for time-stamped samples in epidermis and dermis. Samples collected at different time points are indicated with different point shapes.


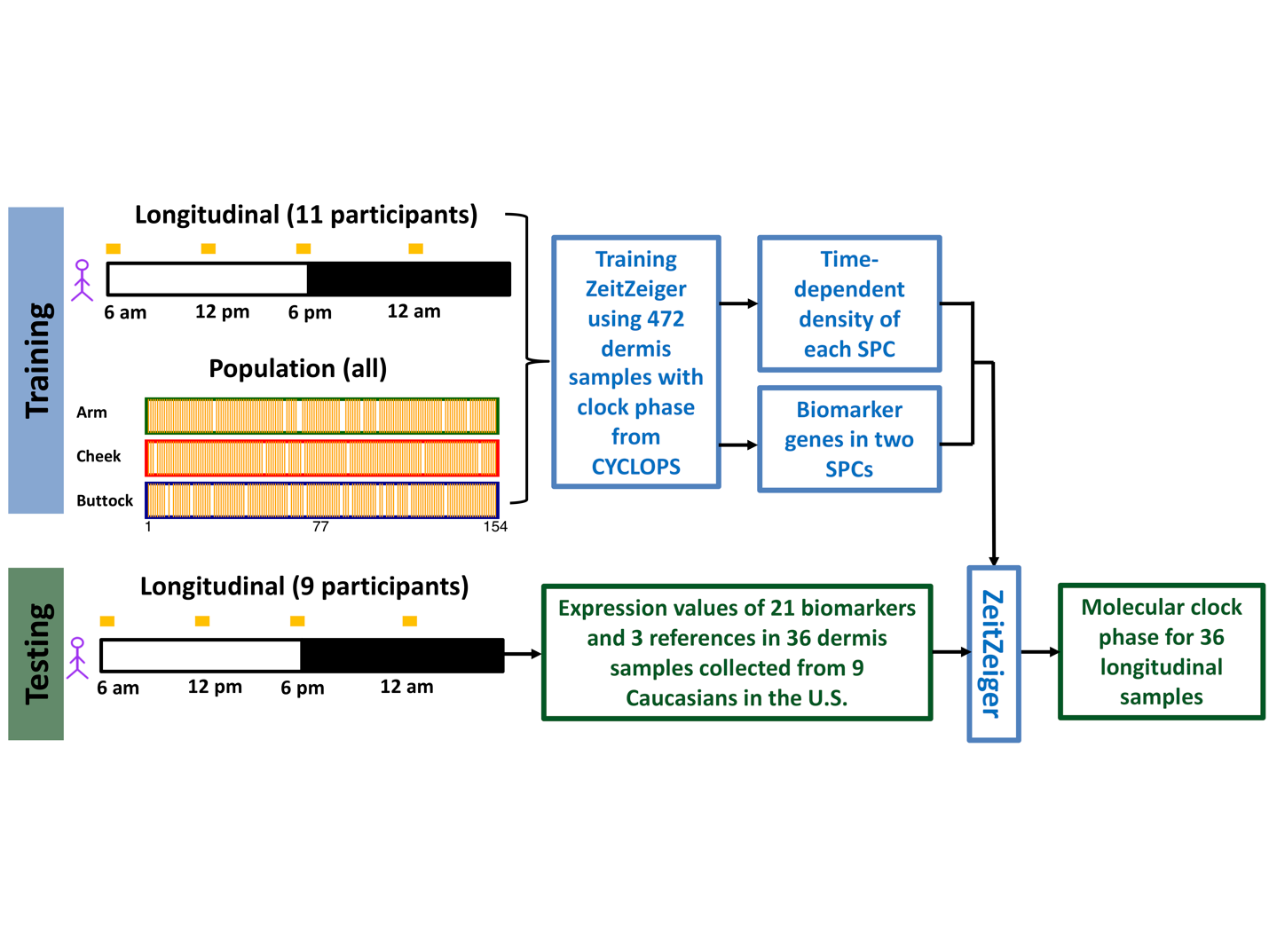


Fig. S6. The pipeline of identifying and testing dermal candidate biomarkers using ZeitZeiger. The training dataset includes 472 longitudinal and population-based dermis samples. Using this training dataset, a set of 21 candidate biomarker genes were selected by ZeitZeiger. The prediction accuracy of candidate biomarkers was further tested using 36 dermis samples collected from 9 male participants in the U.S., and each participant donated one sample at each of four time points (6 AM, 12 PM, 6 PM and 12 AM).


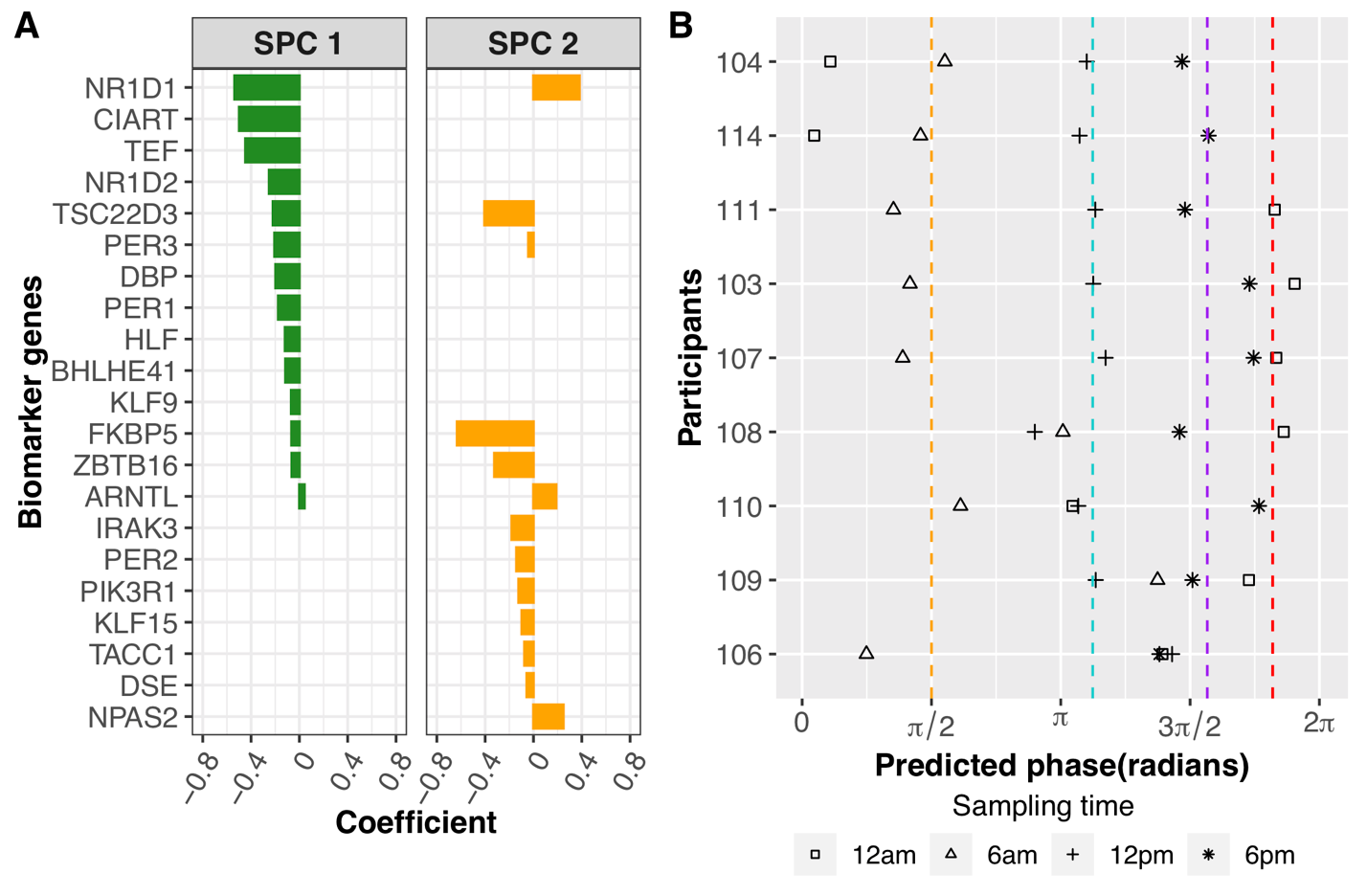


Fig. S7. Candidate biomarkers for predicting molecular clock phase of a single dermal sample. (A) 21 candidate biomarker genes were selected by ZeitZeiger. (B) Validation of candidate biomarkers using dermis samples from 9 participants that were excluded from the training set. The dermis samples were collected every 6 h over a circadian day. Average predicted phases of samples collected at 12 AM, 6 AM, 12 PM and 6 PM are indicated with red, orange, cyan and purple dashed lines, respectively.


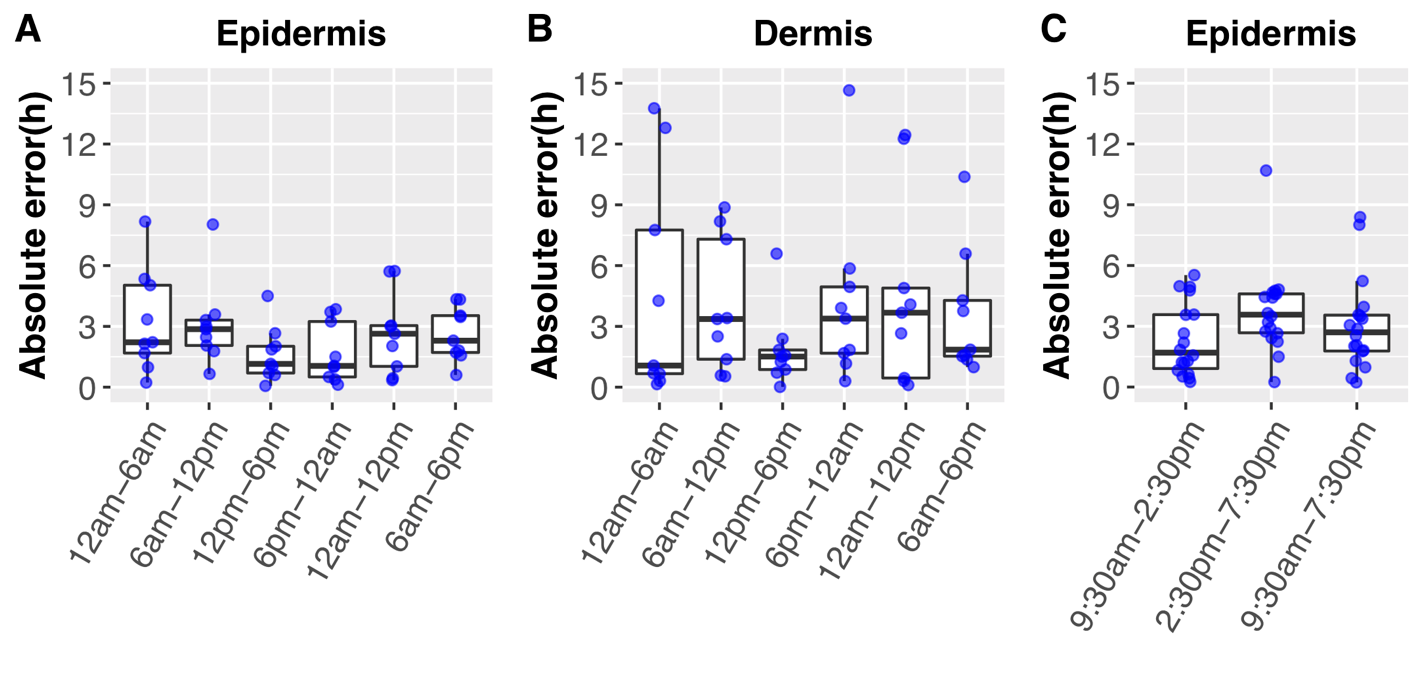


Fig. S8. Prediction accuracy of candidate biomarkers from epidermis and dermis. Absolute errors of phase prediction using selected candidate biomarker genes are plotted for nine participants at six time windows for epidermis (A) and dermis (B). Absolute errors of phase prediction using candidate biomarker genes from epidermis are plotted for 18 participants at three time windows (C) using data from Sporl et al. study (Table S1). The boxes indicate data between the 25th and 75th percentiles with central horizontal lines representing the median values, respectively. The whiskers of boxes show the 5th and 95th percentiles.


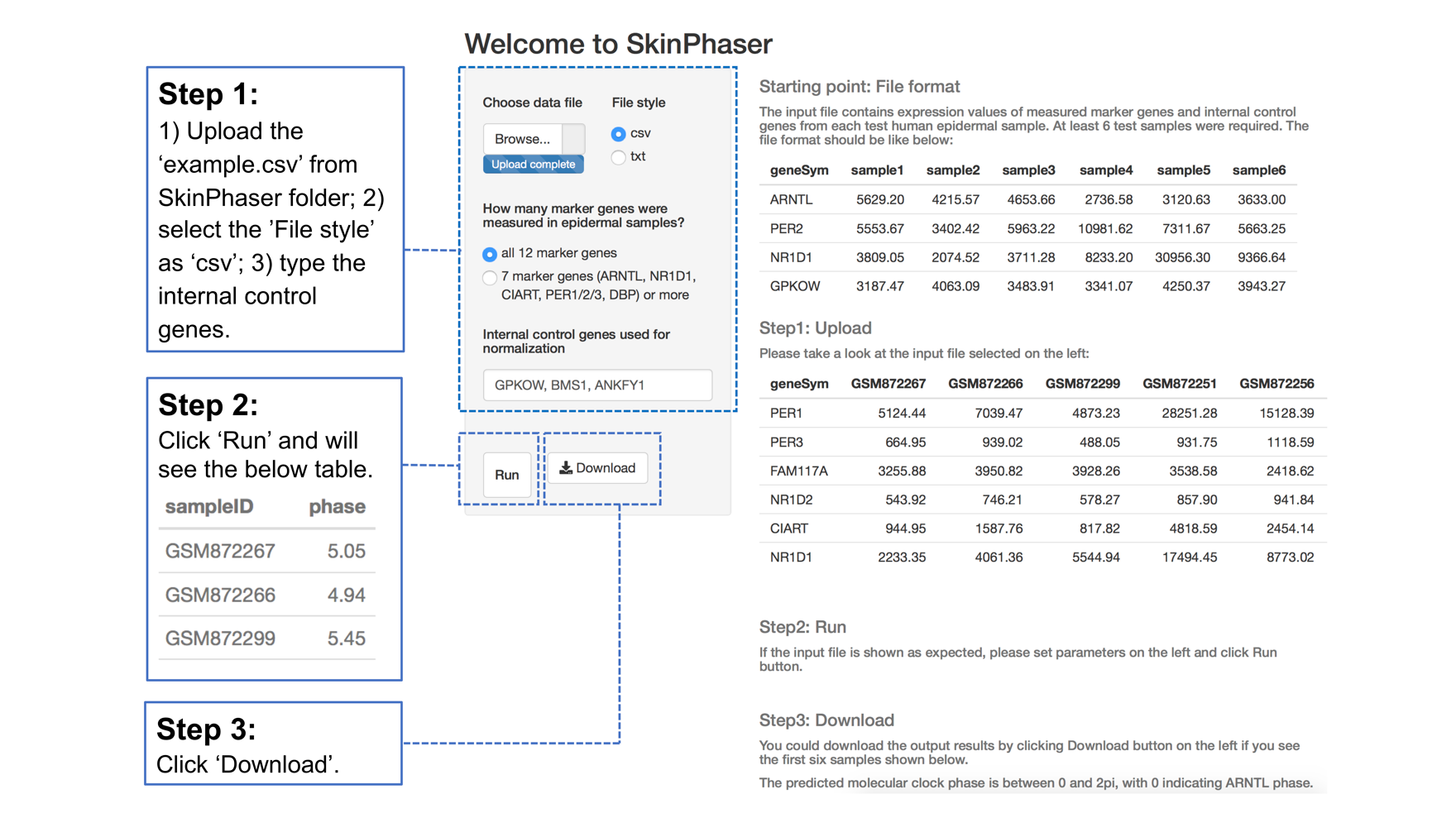


Fig. S9. Three steps of running SkinPhaser. The SkinPhaser app is in GitHub (<https://github.com/gangwug/SkinPhaser>).

**Table S1. The list of skin datasets used in this study**

| **Datasets** | **Reference** | **Platform** | **Site** | **#Samples** | **Experimental design** |
| --- | --- | --- | --- | --- | --- |
| 1. Mouse telogen  (longitudinal) | Geyfman M., et al., 2012, PNAS. GSE38622 | MoGene-1_0-st | telogen | 13 | C57BL/6CR mice were housed under 12 h:12 h LD cycles with food and water ad libitum. Whole skin was collected at 4-h intervals for 48 h. Telogen samples were collected from P46 mice. Equal amounts of RNA from the three mice for each time point were pooled. |
| 2. Epidermal  skin from Caucasian in Germany  (longitudinal) | Sporl F, et al., 2012, PNAS. GSE35635 | Agilent- 014850 | NA | 54 | Suction blisters for 10 male and 10 female volunteers aged 28.35 ± 4.36 y (mean ± SD) were harvested at 9.30 am, 2.30 pm and 7.30 pm. Those 18 participants with 3 samples were selected in this study. |
| 3. Epidermal  skin from Caucasian in the U.S.  (longitudinal) | Wu G., et al., 2018, PNAS.  GSE112660 | HG-219 array | forearm | 79 | Four forearm skin samples were collected at 12am, 6am, 6pm, 12pm for each of 20 male participants, except one missing sample from participant 115. The ages of these 20 participants are between 21 and 49 years old. LCM was performed to separate dermis from epidermis. |
| 4. Dermal  skin from Caucasian in the U.S.  (longitudinal) | This study.  GSE139300 | HG-219 array | forearm | 79 | As described above for dataset 3. |
| 5. Epidermal skin from Caucasian in the U.S. (population) | Kimball A. B., et al., 2018, J Am Acad Dermatol.  GSE139305 | HG-219 array | forearm,  cheek and buttock | 454 | One skin sample from forearm, cheek and buttock was taken from each of 154 female participants, aged between 20 and 74 years old. Samples were designed to collect during the working hours, between 9am to 5pm. LCM was performed to separate dermis from epidermis. There are 17 participants with one missing sample and one participant with two missing samples. |
| 6. Dermal skin from Caucasian in the U.S. (population) | Kimball A. B., et al., 2018, J Am Acad Dermatol.  GSE139305 | HG-219 array | forearm,  cheek and buttock | 452 | As described above for dataset 5. |

**Table S2. The list of software packages used in this study**

| **Software package** | **Version** | **Link** |
| --- | --- | --- |
| affy | 1.62.0 | <https://bioconductor.org/packages/release/bioc/html/affy.html> |
| ape | 5.3 | <https://cran.r-project.org/web/packages/ape/index.html> |
| CircStats | 0.2-6 | <https://cran.r-project.org/web/packages/CircStats/index.html> |
| CYCLOPS | v3.0.2.1 | <https://github.com/gangwug/CYCLOPSv3.0.2.1> |
| julia | 0.3.12 | <https://julialang.org/downloads/oldreleases.html> |
| limma | 3.40.2 | <https://bioconductor.org/packages/3.9/bioc/src/contrib/Archive/limma/> |
| MetaCycle | 1.2.0 | <https://cran.r-project.org/web/packages/MetaCycle/index.html> |
| Oscope | 1.14.0 | <http://bioconductor.org/packages/release/bioc/html/Oscope.html> |
| PSEA | 1.1 | <https://github.com/ranafi/PSEA> |
| R | 3.6.1 | <https://www.r-project.org/> |
| sva | 3.32.1 | <https://bioconductor.org/packages/release/bioc/html/sva.html> |
| zeitzeiger | 2.0.1 | <https://github.com/hugheylab/zeitzeiger> |
